# Frequency of the 7q11.23 inversion polymorphism in transmitting parents of children with Williams syndrome and in the general population does not differ between North America and Europe

**DOI:** 10.1186/1755-8166-4-7

**Published:** 2011-02-28

**Authors:** Colleen A Morris, Carolyn B Mervis, Lucy R Osborne

**Affiliations:** 1 Department of Pediatrics, University of Nevada School of Medicine, Las Vegas, Nevada, USA; 2 Department of Psychological and Brain Sciences, University of Louisville, Louisville, Kentucky, USA; 3 Departments of Medicine and Molecular Genetics, University of Toronto, Toronto, Ontario, Canada

## Abstract

Inversion of the Williams syndrome (WS) region on chromosome 7q11.23 has previously been shown to occur at a higher frequency in the transmitting parents of children with WS than in the general population, suggesting that it predisposes to the WS deletion. Frohnauer et al. recently reported that the frequency of this inversion is not elevated in the parents of children with WS in Germany relative to the German general population. We have compared Frohnauer et al.'s data to those from three previously published studies (Hobart et al., Bayes et al., Osborne et al.), all of which reported a significantly higher rate of 7q11.23 inversion in transmitting parents than in the general population. Results indicated that Frohnauer et al.'s data are consistent with previously reported frequencies of 7q11.23 inversion in North America and Spain in both transmitting parents and the general population.

## 

Dear Editor:

We read with interest the article, "No significantly increased frequency of the inversion polymorphism at the WBS-critical region 7q11.23 in German parents of patients with Williams-Beuren syndrome as compared to a population control" [1]. The human chromosome 7q11.23 region is prone to genomic rearrangement, with deletion leading to the neurodevelopmental disorder, Williams syndrome (WS) [2]. Inversion of the region also occurs but has no clinical effect [3]. We, and others, have previously reported that rates of 7q11.23 inversion in WS progenitors are between four and five times higher than in the general population, suggesting that the inversion predisposes the chromosome to subsequent deletion [4-6]. Frohnauer et al. present their study of German families with WS and conclude that in this population the rate of inversion is not elevated compared to non-WS families [1]. Our review of their study results in a different conclusion than the authors': We find that their data *agree *with previously published reports of the frequency of inversion in Williams syndrome (WS) progenitors [4-6] and in the general population [4-6]. The major difference in their data analysis was that they treated the couple as a unit, rather than each parent as an individual contributing one chromosome 7 to the offspring. For instance, Frohnauer et al. report that 7/51 couples not having a child with WS had one spouse with an inversion [1]. The rate of inversion for non-transmitting parents, then, is 7/102 (6.9%), not 7/51 (13.8%). A binomial comparison of this proportion (7/102) to the 5.8% rate (15/257 non-transmitting parents) reported in Hobart et al. [3] for a much larger sample yields exact p = .76.

Frohnauer et al. further report that in 5/24 couples who had a child with WS, one (4/24 couples) or both (1/24 couples) parents had an inversion [1]. Therefore, 6/48 parents had an inversion. The authors determined the parent of origin for the deletion only for the one patient who had an inverted chromosome; in this case, the father transmitted the inverted chromosome and the mother, who did not have an inversion, was the parent of origin of the deletion. If the transmitting parent in the other four couples had an inversion, then the rate of inversion in transmitting parents in this sample was 16.7%. A binomial comparison of this proportion (4/24) to the probability reported in Hobart et al. [4] (64/257 = 24.9%) yields exact p = .50.

Thus, in contrast to Frohnauer et al.'s [1] claim, the findings from their study and Hobart et al. [4] are consistent both with regard to the rate of 7q11.23 inversion in the general population and the rate of 7q11.23 inversion among transmitting parents of children with WS, where the inversion is a risk factor for having a child with WS. Frohnauer et al.'s rate of inversion in transmitting parents of children with WS also is consistent with that reported by Bayes et al. [5] for a Spanish sample (n = 74; binomial p = .31)^1 ^and Osborne et al. [6] for a North American sample (n = 12; Fisher's exact test p = .40). Furthermore, Frohnauer et al.'s rate of inversion in the German general population is consistent with that reported by Bayes et al. for the Spanish general population (binomial p = .73) and Osborne et al. for the North American general population (Fisher's exact test p = .19). In conclusion, Frohnauer et al. [1] have replicated prior inversion-rate findings from both Spanish [5] and North American populations [4,6] in the German population.

## Footnote

^1^Frohnauer et al. [1] argued that although Bayes et al. [5] reported a 28% rate of inversion in transmitting parents based on SSN assays, the actual rate as confirmed by three-color FISH was 5.5%, which was almost identical to the 5.7% rate Bayes et al. found for nontransmitting parents. However, the 5.5% figure is based on Frohnauer et al.'s incorrect assumption that three-color FISH was used to test all 74 transmitting parents and that the inversion was confirmed in only 4/74 (5.5%). In fact, Bayes et al. stated that they performed three-color FISH for only four of the parents who had been shown based on SSN assays to have an inversion and the FISH tests confirmed the inversion for all four, supporting the validity of the 28% inversion rate obtained using the SSN assay method.

## Abbreviations

WS: Williams syndrome

## Competing interests

The authors declare that they have no competing interests.

## Authors' contributions

CAM, CBM and LRO conceived the study. CBM performed the statistical analysis. CAM, CBM and LRO drafted the manuscript. All authors read and approved the final manuscript.
